# Ovarian T-cell lymphoma in a dog with chronic protein-losing enteropathy

**DOI:** 10.1186/s13028-025-00847-0

**Published:** 2026-01-27

**Authors:** Jacob Kvesel Mortensen, Emil Wikström, Norbert van de Velde

**Affiliations:** 1 Gothenburg Animal Hospital Evidensia, Produktvägen 5, 435 33 Mölnlycke, Sweden; 2 Department of Pathology and Wildlife Diseases, Swedish Veterinary Agency, Ulls väg 2B, 751 89 Uppsala, Sweden

**Keywords:** Ascites, Canine, Extranodal lymphoma, Ovarian neoplasia

## Abstract

**Background:**

Lymphoma is one of the most common malignancies in dogs, but ovarian lymphoma is exceedingly rare, with only a few reported cases. Most canine lymphomas are of B-cell origin, whereas T-cell lymphomas are generally associated with a more aggressive clinical course. Protein-losing enteropathy (PLE) is a well-documented cause of hypoalbuminemia in dogs, often resulting from lymphangiectasia or chronic enteropathies. The potential relationship between chronic immune-mediated disease and the development of lymphoma remains unclear.

**Case presentation:**

A 10-year-old intact female Miniature Poodle presented with chronic diarrhea, ascites and hypoalbuminemia. Abdominal ultrasound revealed severe intestinal mucosal striation, consistent with PLE, but no evidence of neoplasia. The dog was treated with methylprednisolone, chlorambucil, and cyclosporine, achieving long-term clinical stability. Fourteen months after initial presentation, the dog developed acute gastrointestinal signs, and ultrasound revealed an ovarian mass. Cytology, histopathology, and immunohistochemistry confirmed a diagnosis of ovarian T-cell lymphoma. No other neoplastic lesions were detected at that time raising the possibility of a primary ovarian origin, though widespread disease at necropsy suggests secondary involvement. The dog remained clinically stable for five months after lymphoma diagnosis but ultimately developed widespread disease, including hepatic, splenic, and adrenal involvement, leading to euthanasia. Necropsy confirmed multi-organ dissemination of the T-cell lymphoma, while histopathology of the intestines revealed signs of chronic lymphangiectasia but no definitive diagnosis of intestinal lymphoma.

**Conclusions:**

This case highlights the diagnostic challenges of extranodal lymphoma in dogs and the importance of considering neoplasia in cases of chronic protein-losing enteropathy. The potential role of chronic inflammation and long-term immunosuppressive therapy in lymphomagenesis remains unclear and warrants further study.

## Background

 Lymphoma represents one of the most common malignancies in dogs and comprises 83% of all canine hematopoietic malignances [1, 2]. Most canine lymphomas are of B-cell origin and primarily affect peripheral lymph nodes, although extranodal presentations are also well-documented. The gastrointestinal tract is a common extranodal site, often associated with protein-losing enteropathy (PLE) due to intestinal lymphangiectasia and mucosal disruption. PLE in dogs can lead to profound hypoalbuminemia and clinical manifestations such as diarrhea, weight loss, and ascites. Despite extensive documentation of lymphoma in various anatomical locations, ovarian lymphoma is exceedingly rare in dogs, with only a few cases described in the veterinary literature [3–5].

Ovarian tumors in dogs are typically epithelial, stromal, or germ-cell in origin, while primary lymphoid neoplasms of the ovaries are an infrequent finding. The clinical presentation of ovarian lymphoma may be nonspecific, with abdominal distension, vaginal discharge, or systemic signs such as lethargy and anorexia. Diagnosis requires advanced imaging and cytological or histopathological examination. Immunostaining is often necessary to confirm the subtype of lymphoma, as T-cell lymphomas tend to exhibit more aggressive biological behavior and poorer prognosis compared to B-cell lymphomas [1].

This report describes a unique case of a Miniature Poodle diagnosed with an ovarian T-cell lymphoma following a prolonged clinical course of chronic PLE. The case highlights the diagnostic challenges, the therapeutic considerations for concurrent lymphoma and PLE, and the clinical outcome. This is one of the first documented cases of ovarian T-cell lymphoma associated with chronic protein-losing enteropathy in veterinary medicine.

## Case presentation

A 10-year-old intact female Miniature Poodle was referred to Gothenburg Animal Hospital Evidensia from another clinic on 31 August 2022 because of chronic diarrhea and abdominal swelling. Prior to presentation, the dog had experienced worsening diarrhea and was given a commercial gastrointestinal diet. The dog received no medications at the time of presentation.

At the time of admission, the dog weighed 5.2 kg and had a normal body condition score. During the physical exam, the dog was bright, alert, and responsive; had pink and moist mucous membranes; no alteration on cardiopulmonary auscultation; ascites; and no vaginal discharge. Blood work revealed hypoalbuminemia of 13 (22–39 g/L), hypoproteinemia of 40 (52–82 g/L), hypocholesterolemia of 2.57 (2.84–8.26 mmol/L), thrombocytosis of 686 (148–484 K/µL), hypofolatemia of 20.3 (21.1–54 nmol/L), and hypocobalaminemia of 145 (173–599 pmol/L). Ultrasound of the abdomen revealed severe ascites and a severely striated mucosal layer of the duodenum and proximal and central jejunum. There were small cysts in the endometrium, and other abdominal organs were unremarkable. Normal liver and kidney values (alanine transaminase (ALT), alkaline phosphatase (ALP), glucose, urea and creatinine) in conjunction with ultrasound findings did not raise a suspicion of urinary loss or liver failure as a cause of hypoalbuminemia. Abdominocentesis of the ascites revealed a transudate with a density of 1.005. These findings lead to a presumptive diagnosis of protein-losing enteropathy with secondary hypoalbuminemia and ascites, even though no intestinal biopsy was taken. The dog was started on treatment with methylprednisolone 0.8 mg/kg twice daily with gradual tapering, rivaroxaban 1 mg/kg once daily, hydrolyzed diet, and probiotics.

One week after initial presentation, the ascites had resolved, the dog had lost 0.9 kg due to the resolution of ascites, feces were normal, and serum albumin had increased to 22 (22–39 g/L). Rivaroxaban was discontinued, and vitamin B supplementation was initiated for the dog.

Three weeks after initial presentation, the dog was still in remission with no change in body weight, serum albumin of 23 (22–39 g/L), and thrombocyte levels within the reference range.

Six weeks after initial presentation, the dog remained in remission, serum albumin was 23 g/L, and chlorambucil (2 mg twice weekly) was initiated to improve serum albumin levels while tapering the methylprednisolone dose.

Eight weeks after initial presentation, side effects of methylprednisolone developed, including a dry fur coat, mild alopecia, and mild muscle mass reduction.

Approximately three months after presentation, serum albumin peaked at 26 g/L, and serum cobalamin and folic acid had normalized, allowing discontinuation of cobalamin and folic acid supplementation. Under chlorambucil monotherapy, serum albumin stabilized between 24 and 26 g/L for several months.

Approximately 12 months after initial presentation, the dog presented with acute nausea and decreased appetite. Clinical examination revealed a quiet, alert, and responsive demeanor, nausea and a moderately painful abdomen on palpation. Serum albumin had decreased to 21 g/L. Ultrasound of the abdomen revealed no further findings as compared to prior ultrasound examinations that could explain the symptoms. After supportive hospitalization, the dog’s appetite improved, and nausea resolved. Cyclosporine (5 mg/kg once daily) was added to the treatment regimen due to the relapse of symptoms associated with the PLE and the declining serum albumin.

Approximately 14 months after presentation, the dog was clinically stable with a serum albumin of 23 g/L, and serum cobalamin and folic acid were within reference ranges.

**Fig. 1:**
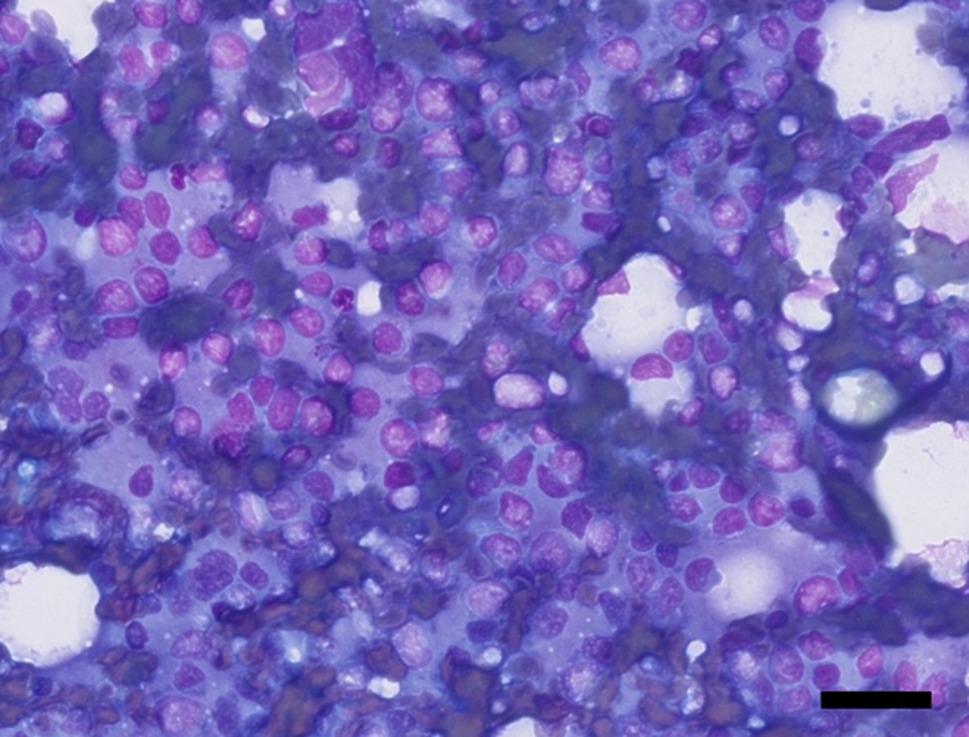
Cytology of ovarian mass demonstrating malignant lymphoid population. Cytology showing a monomorphic population of round cells consistent with lymphocytes, exhibiting high nuclear-to-cytoplasmic ratios and irregular nuclei with finely stippled chromatin. Interpreted as a round cell neoplasia consistent with large cell lymphoma. Hemacolor^®^ stain, 40x (scalebar represents 50 μm)

Later in the same month, the dog presented with acute watery diarrhea and decreased appetite. On examination, the dog was bright, alert, and responsive, with a temperature of 38.3 °C, pink and slightly dry mucous membranes, unremarkable cardiopulmonary auscultation, abdominal pain, and mild transparent, odorless vaginal discharge. Ultrasound revealed a 2.2 × 1.6 cm isoechoic ovarian mass with a moderately hyperechoic surrounding area. No changes were noted in other abdominal organs compared to prior ultrasounds, and lung ultrasound was unremarkable. Fine-needle aspiration and cytological examination of the ovarian mass yielded a smear that was moderately hemodiluted but of overall good diagnostic quality with a moderate number of nucleated cells (Fig. 1). The smear was dominated by a monomorphic population of round cells, most consistent with lymphocytes. These cells measured approximately 10 μm in diameter and exhibited moderate to high nuclear-to-cytoplasmic ratios. Nuclei were generally round but often irregular, showing indentations or convolutions, with finely stippled chromatin. Nucleoli were not apparent. The cytoplasm was scant to moderate in amount, pale, and only rarely vacuolated. Mitotic figures were not a prominent feature. No ovarian stromal or epithelial elements were identified to confirm direct ovarian origin. The findings were interpreted as a round cell neoplasia consistent with a large cell lymphoma. Fine-needle aspiration of the liver was also performed and there was sufficient evidence to confirm sampling of the liver parenchyma, but otherwise the sample was limited because of low cellular yield and a large amount of blood. There was no clear indication for round cell neoplasia in the liver although it could not be confidently excluded because of the limitations of the sample. The dog’s clinical condition improved the following day after supportive hospitalization.

Ovariohysterectomy was performed three days later for therapeutic purposes and confirmation of the diagnosis. The left ovary was grossly enlarged (3.0 × 3.5 cm) and located adjacent to the kidney. The entire ovary was excised, but without a possible margin. The right ovary and other abdominal organs appeared macroscopically normal. The left ovary was submitted for histological evaluation which revealed that the entire ovarian tissue was obliterated by a cell-rich, invasive neoplasm of small well-defined round cells showing high mitotic activity (40 mitoses per 10 HPF – 2.37 mm^2^) diagnosed as T-cell lymphoma. Subsequent immunohistochemical staining was performed to confirm and further differentiate the diagnosis. There was almost uniform cytoplasmic staining of the neoplastic cells with CD3 antibodies which stains normal and neoplastic T-cells, confirming this tumour as a T-cell lymphoma. Chlorambucil and cyclosporine treatment was continued with no additional chemotherapy initiated. However, a doxorubicin-based protocol was considered and discussed with the owner because lymphoma is fundamentally considered a systemic malignancy rather than a strictly localized one and because the ovarian tumor could not be excised with a margin. On the other hand, because of the lack of solid evidence to suggest the exact advantage of adjuvant doxorubicin-based chemotherapy compared with ovariohysterectomy alone, particularly for ovarian high-grade lymphoma, the continuation of chlorambucil and cyclosporine was chosen to prevent relapse of the PLE. However, a possible relapse of the ovarian lymphoma before recurrence of PLE-related symptoms remained a concern.

Follow-up ultrasound for restaging purpose performed five weeks later revealed no morphologic abnormalities in the abdominal organs.

Four months after the lymphoma was diagnosed, the dog presented with mild to moderate acute abdominal pain. Blood work showed elevated C-reactive protein (CRP) (80 mg/L, reference 0–10 mg/L), a positive canine pancreas-specific lipase SNAP test, and serum albumin of 20 g/L. Abdominal ultrasound revealed multiple enlarged cranial abdominal lymph nodes (8–10 mm) and a 10 × 14 mm mass on the right adrenal gland, while the pancreas appeared morphologically unremarkable. The clinical signs resolved the next day after supportive treatment at home. No further investigation of the adrenal mass and possible pituitary- or adrenal-dependent hyperadrenocorticism was made at this point, as no clinical signs such as polyuria/polydipsia, polyphagia, or pendulous abdomen were present. The findings were assessed as a possible neoplastic relapse in the right cranial abdominal cavity, and the positive SNAP test result was regarded as inconclusive for diagnosing pancreatitis in the absence of ultrasonographic pancreatic changes.

Despite intermittent mild diarrhea and decreased appetite, the dog improved clinically over the next few weeks, with normalized CRP and serum albumin of 24 g/L.

**Fig. 2:**
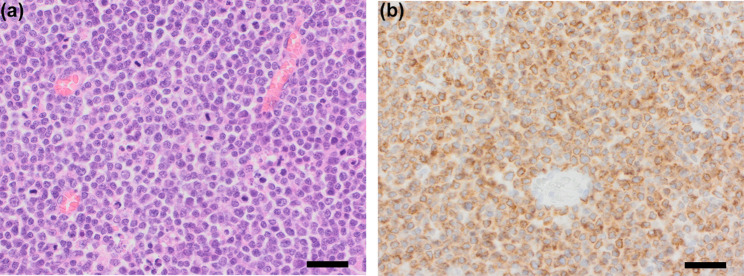
**a**. Ovary replaced by densely cellular invasive lymphoid neoplasm. The entire ovarian tissue is obliterated by a cell-rich, invasive neoplasm of sheets of small well-defined tumor cells showing high mitotic activity. Hematoxylin & Eosin stain, 40x (scalebar represents 50 μm). **b**. Ovarian neoplastic cells show strong CD3 immunoreactivity. Strong and almost uniform brown cytoplasmic staining of the neoplastic cells was seen, confirming T-cell lymphoma. Immunohistochemical stain for CD3, 40x (scalebar represents 50 μm)

**Fig. 3:**
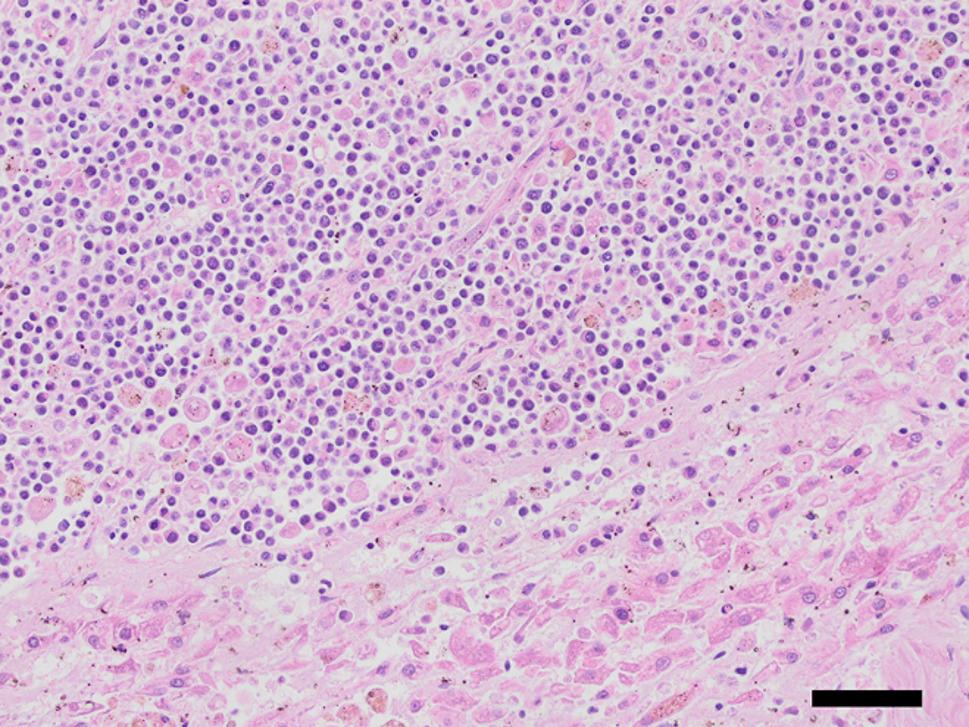
Hepatic tissue extensively infiltrated by malignant lymphoid neoplasm. The hepatic parenchyma is largely obliterated by a cell-rich, invasive neoplasm of sheets of small well-defined tumor cells showing high mitotic activity. The neoplasm is surrounded by degenerated hepatic parenchyma. Hematoxylin & Eosin stain, 20x (scalebar represents 100 μm)

**Fig. 4:**
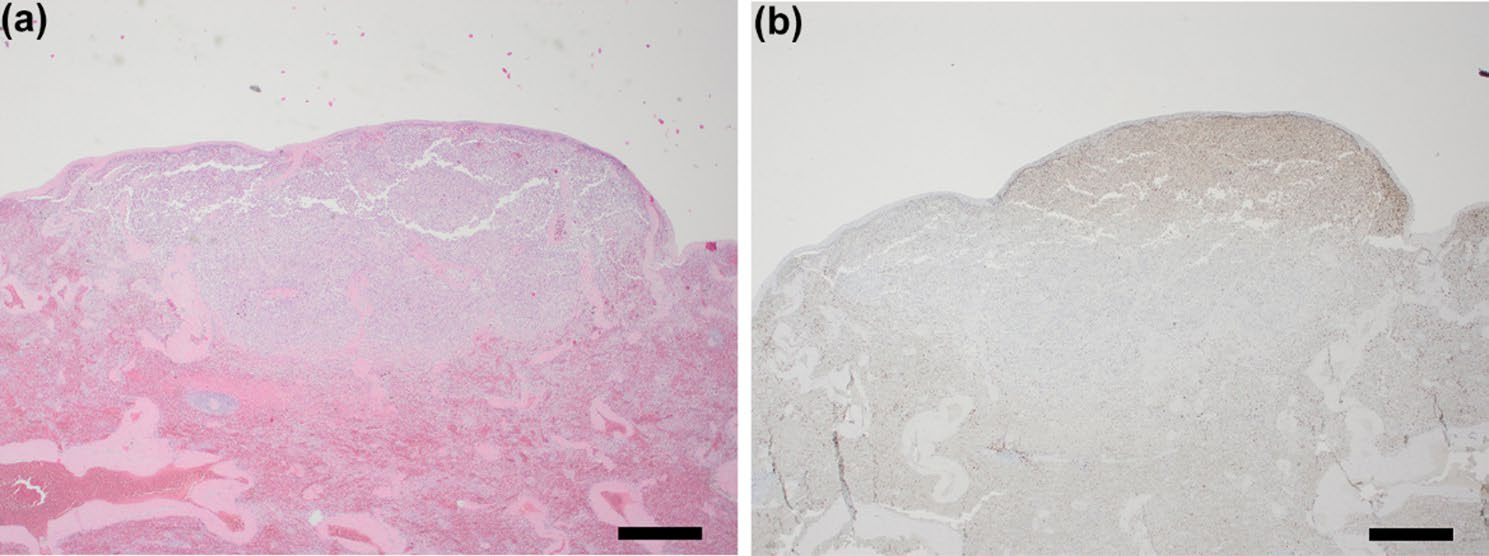
**a**. Splenic capsule underlain by infiltrative lymphoid neoplasm. Within the spleen, a cell-rich, invasive neoplasm was seen directly under the splenic capsule. Hematoxylin & Eosin stain, 2x (scalebar represents 1 mm). Inset: The cells have a moderate amount of eosinophilic cytoplasm and large round oval nuclei filled with finely granular to pale chromatin with often a clear eosinophilic nucleus. Hematoxylin & Eosin stain, 40x (scalebar represents 50 μm). **b**. Splenic neoplastic cells exhibit positive CD3 immunostaining. The neoplastic cells stain positively (brown) for CD3. Immunohistochemical stain for CD3, 2x (scalebar represents 1 mm)

**Fig. 5:**
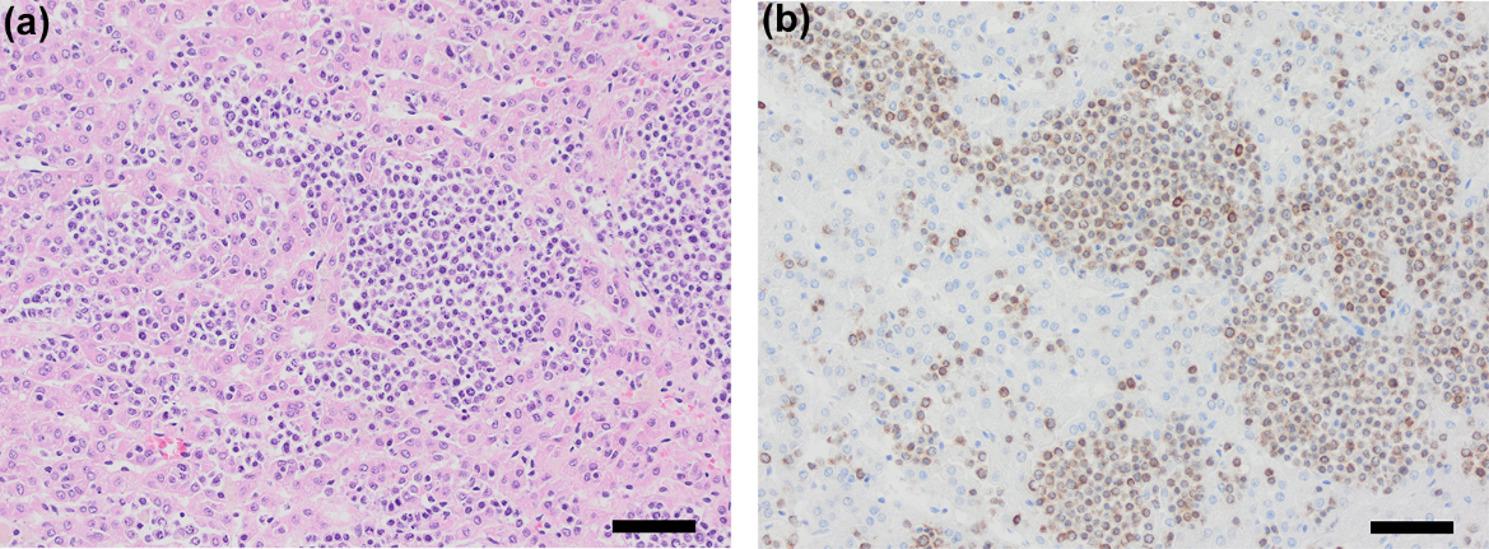
**a**. Adrenal gland infiltrated by small neoplastic lymphoid cells. Infiltrates of small basophilic neoplastic cells were seen between the trabeculae of normal adrenal gland tissue (large eosinophilic cells). Hematoxylin & Eosin stain, 20x (scalebar represents 100 μm). **b**. Adrenal neoplastic cells show strong CD3 positivity. The neoplastic cells stain positively (brown) for CD3. Immunohistochemical stain for CD3, 20x (scalebar represents 100 μm)

Approximately 19 months after initial presentation, the dog experienced a relapse of ascites, appeared lethargic, but continued to eat and drink. Examination revealed ascites, pink and sticky mucous membranes, and unremarkable cardiopulmonary and peripheral lymph node examinations. Ultrasound confirmed ascites and enlarged abdominal lymph nodes (maximum size 1.4 × 0.7 cm). The dog was euthanized on 11 April 2024, 5 months after the lymphoma diagnosis. A timeline with a summary of the case is presented in Table 1; Figs. 2, 3, 4 and 5 depict the histopathologic findings.

**Table 1:** Timeline of clinical signs, treatments, and outcomes in a dog with protein-losing enteropathy that later developed T-cell lymphoma

Time after initial presentation	Event
Week 0 (Initial Presentation)	Dog presented with chronic diarrhea, ascites, and hypoalbuminemia. PLE treatment was initiated.
Week 1	Ascites resolved, feces normalized, and serum albumin improved. Rivaroxaban discontinued.
Week 6	Chlorambucil added while tapering methylprednisolone.
Month 3	Serum albumin peaked at 26 g/L. Chlorambucil monotherapy maintained albumin levels.
Month 12	Relapse of PLE symptoms with nausea and appetite loss. Cyclosporine added.
Month 14	Acute diarrhea and ovarian mass detected. Cytology confirmed lymphoma. Ovariohysterectomy performed. Histopathology and immunohistochemistry confirmed T-cell lymphoma. Chlorambucil and cyclosporine continued.
Month 18	Possible neoplastic relapse with enlarged abdominal lymph nodes. Supportive care improved symptoms.
Month 19	Relapse of ascites, lethargy, and disease progression. Ultrasound confirmed progression. Dog euthanized. Necropsy confirmed multi-organ spread of T-cell lymphoma.

At necropsy and histological examination, numerous neoplastic lesions were found in a variety of organs including liver, spleen, and the right adrenal gland. In the liver, large infiltrates of small to moderately sized, individualized, round to oval cells with a small amount of eosinophilic cytoplasm and a small round nucleus with granular chromatin markedly disrupted the hepatic parenchyma and showed a mitotic count of approximately 6 per 10 HPF (2,37 mm^2^) (Fig. 2). Within the spleen, a single subcapsular nodule was seen, which existed histologically of an accumulation of individual, round to oval, moderately sized cells (Fig. 3a). The cells had a moderate amount of eosinophilic cytoplasm and large round oval nuclei filled with finely granular to pale chromatin with often a clear eosinophilic nucleus (Fig. 3a, inset). The number of mitoses was approximately 8 per 10 HPF (2,37 mm^2^). In the right adrenal gland, large infiltrates of moderate to large polygonal cells with moderate amounts of eosinophilic cytoplasm and large round nuclei partially obliterated the adrenal cortex and medulla. Anisokaryosis and -cytosis was moderate and the number of mitoses was around 16 per 10 HPF (2,37 mm^2^) (Fig. 5a). The appearance of the neoplastic cells gave an initial impression of a carcinoma (epithelial malignant tumor) in the spleen and adrenal gland, but immunohistochemical staining on the spleen and adrenal gland revealed the presence of CD3-positive tumor cells (Figs. 3b and 5b), which confirmed that all neoplastic lesions found at necropsy were caused by the multi-organ spread of this T-cell lymphoma. Assessment of the intestine was limited by autolysis which made detailed assessment difficult, but obvious signs of neoplasia were not seen. However, dilated lymphatic spaces in the intestinal villi indicating lymphangiectasia were seen during histological examination of the intestine and could be an explanation for the diarrhea.

## Discussion and conclusions

Lymphoma is a common malignancy in dogs, but ovarian lymphoma is rare, with only a few reported cases [3–5] like findings in humans. This report describes a case of ovarian T-cell lymphoma in a dog with chronic PLE. The dog, treated for PLE with immunosuppressive drugs, remained clinically stable for over a year before an ovarian mass was detected. Diagnosis was confirmed through cytology, histopathology, and immunohistochemistry.

We considered whether the ovarian lymphoma was primary or secondary. Primary ovarian lymphoma in dogs has only been described once [3], whereas secondary has been reported twice [4, 5]. Given the widespread neoplastic infiltration at necropsy, including the spleen, liver, and adrenal gland, the lymphoma was most likely secondary. However, no other organ involvement was detected at the time of diagnosis, raising the possibility that the ovary was the primary origin. Without earlier histopathologic confirmation of lymphoma elsewhere, definitive determination of the site of origin remains uncertain.

Human and canine lymphoma share multiple similarities. In both species, non-Hodgkin lymphoma accounts for most cases and most present as multicentric disease [6]. B-cell origin is more common than T-cell, with the latter generally associated with a poorer prognosis. Standard therapy typically involves multiagent chemotherapy protocols. Ovarian involvement is also very uncommon in women, with primary tumors being less frequent than secondary [7, 8]. Among these, diffuse large B-cell lymphoma predominates, and diagnosis relies on histopathology and immunophenotyping, including CD20 and CD79a expression [7–9]. Multiagent chemotherapy is first line treatment, while surgery is primarily diagnostic [6, 7]. In contrast to the predominance of B-cell phenotype in humans, the present case represented a T-cell phenotype. However, given the extreme rarity of primary ovarian lymphoma in dogs, it is not possible to determine whether our finding of a T-cell lymphoma represents a true species-specific difference or simply the rarity of reported cases, and further studies are needed to clarify potential similarities and differences in disease progression and treatment response.

The concurrent chronic PLE and development of ovarian lymphoma in this case leads to the speculation whether there could be a causal relationship between these diseases or whether they were independent comorbidities. We considered whether chronic inflammation associated with the PLE could predispose for the development of the lymphoma. Chronic inflammation in humans has been linked as an important risk factor to the development of lymphoma [10]. While certain autoimmune reactions and chronic infections in humans seem to be the most common inflammatory triggers for chronic inflammation in human lymphomagenesis there is sparse evidence showing that the same applies to dogs. An example of this sparse evidence is the link between immune mediated thrombocytopenia and a higher risk of developing lymphoma when compared to the normal dog population [11]. However, no risk of subsequent lymphoma development could be linked to multiple other immune mediated diseases in the same study. For chronic infections, Epstein-Barr virus has been linked to some forms of human lymphoma but was not associated with the development of lymphoma in dogs [12]. Therefore, further research is needed to identify specific causes of chronic inflammation that could lead to the development of ovarian lymphoma.

Besides chronic inflammation, we also considered whether long-term treatment with chlorambucil could have contributed to the subsequent development of ovarian lymphoma. Chlorambucil is an alkylating agent that has been linked to secondary malignancies like leukemia and myelodysplastic syndromes due to DNA damage in humans [13]. However, the association between chlorambucil and the development of lymphomas, such as ovarian lymphoma, is less clear. While alkylating agents have been linked to secondary hematologic malignancies, the evidence primarily pertains to leukemias rather than lymphomas [14]. In dogs, treatment with a chlorambucil-prednisolone combination is commonly described for protein-losing enteropathy, especially to mitigate side effects of prednisolone monotherapy and in refractory cases, and chlorambucil appears to be superior to azathioprine, another immunosuppressive alternative, in terms of efficacy [15, 16]. Therefore, although chlorambucil treatment carries a recognized risk for certain secondary cancers in humans, current evidence does not clearly support a causal relationship between chlorambucil therapy and the subsequent development of lymphoma in dogs. Nonetheless, its widespread use in canine PLE highlights the clinical relevance of considering this potential risk.

A limitation of the current study is the lack of an intestinal biopsy prior to starting immunosuppressive therapy to confirm an underlying reason for the PLE. The reason for not performing a biopsy prior to therapy was unfortunately not documented but might have been influenced by anesthesia risk associated with hypoalbuminemia. Biopsy was, however, considered later but not performed based on a risk-benefit analysis because the PLE was already in remission. In addition, clonality testing (PCR for Antigen Receptor Rearrangements) was not considered at the time; such testing might have provided further insight into whether gastrointestinal lymphoma was present earlier in the disease course. In many cases ruling out urinary loss and liver failure as causes to hypoalbuminemia is enough to start treatment with immunosuppressive drugs and resolution of clinical signs and normalization of serum albumin is usually achieved within few days. In this case urinary loss and liver failure were not suspected, based on normal ALT, ALP, glucose, urea and creatinine. Optimally, urine protein-to-creatinine ratio should have been measured to exclude urinary loss. In addition, normal pre- and postprandial bile acids would have excluded liver failure. Considering the possibility of intestinal lymphoma this could have been suspected if cytology was performed on the ascites in case inflammatory cells or neoplastic cells were seen. Altogether, treatment response and clinical course indicate that PLE is most likely as an underlying cause to the hypoalbuminemia and do not raise suspicion of either urinary protein loss or liver failure. The autopsy findings particularly support lymphangiectasia as being the underlying reason to the PLE.

This case report describes a rare presentation of ovarian T-cell lymphoma in a dog with chronic PLE. The prolonged clinical stability of the PLE before lymphoma diagnosis underscores the complexity of managing concurrent immune-mediated and neoplastic diseases. The absence of detectable neoplastic lesions at the time of ovarian lymphoma diagnosis raises uncertainty about whether it was primary or secondary, though widespread dissemination at necropsy supports the latter.

This case highlights the diagnostic challenges associated with extranodal lymphoma and the need for a thorough workup in dogs with chronic gastrointestinal disease and hypoalbuminemia. Additionally, it raises questions regarding potential links between chronic inflammation, chronic immunosuppressive therapy, and lymphoma development, warranting further investigation.

Future research on the relationship between chronic PLE, chronic immunosuppressive therapy and lymphoid malignancies in dogs may provide deeper insights into pathogenesis and treatment strategies.

## Data Availability

The datasets used and/or analyzed during the current study are available from the corresponding author on reasonable request.

## Abbreviations

ALP: Alkaline phosphatase
ALT: Alanine transaminase
CRP: C-reactive protein
PLE: Protein-losing enteropathy

## Declarations

### Prior publication

Data have not been published previously.
